# Comparison of Oxidative Status of Human Milk, Human Milk Fortifiers and Preterm Infant Formulas

**DOI:** 10.3390/foods8100458

**Published:** 2019-10-08

**Authors:** Luisa Pozzo, Simona Cirrincione, Rossella Russo, Magdalena Karamać, Ryszard Amarowicz, Alessandra Coscia, Sara Antoniazzi, Laura Cavallarin, Marzia Giribaldi

**Affiliations:** 1 Institute of Agricultural Biology and Biotechnology, National Research Council, 56124 Pisa, Italy; luisa.pozzo@ibba.cnr.it (L.P.); rossella.wilbur@gmail.com (R.R.); 2 Institute of Sciences of Food Production, National Research Council, 10095 Grugliasco (TO), Italy; sara.antoniazzi@ispa.cnr.it (S.A.); laura.cavallarin@ispa.cnr.it (L.C.); 3 Institute of Animal Reproduction and Food Research, Polish Academy of Sciences, 10748 Olsztyn, Poland; m.karamac@pan.olsztyn.pl (M.K.); r.amarowicz@pan.olsztyn.pl (R.A.); 4 Neonatal Intensive Care Unit, University of Torino, Città della Scienza e della Salute, 10126 Torino, Italy; alessandra.coscia@unito.it; 5 Research Center for Engineering and Agro-Food Processing, Council for Agricultural Research and Economics, 10035 Torino, Italy; marzia.giribaldi@crea.gov.it

**Keywords:** donkey milk, human milk, infant formulas, protein fortifiers, malondialdehyde, TEAC

## Abstract

Preterm and low birth weight infants require specific nutrition to overcome the accumulated growth deficit, and to prevent morbidities related to postnatal growth failure. In order to guarantee an adequate nutrient-intake, mother’s own milk, when available, or donor human milk, are usually fortified with additional nutrients, in particular proteins. Fortification with processed ingredients may result in additional intake in oxidative compounds, deriving from extensive heat treatments, that are applied during processing. The aim of the present work was to compare the in vitro antioxidant activity and oxidative compound content conveyed by different preterm infant foods and fortifiers, namely raw and pasteurized human milk, two different preterm infant formulas, three bovine milk-based fortifiers and two experimental donkey milk-based fortifiers. Univariate and multivariate statistical analyses revealed significant differences between the different products. The use of human milk minimizes the intake of dietary oxidative compound in comparison to infant formulas, irrespective of pasteurization or fortification, especially as far as malondialdehyde content is concerned. The addition of fortifiers to human milk increases its antioxidant capacity, and the choice of the protein source (hydrolysed *vs.* whole proteins) differently impacted the resulting total antioxidant capacity of the diet.

## 1. Introduction

Preterm infants, including extremely and very low birth weight (LBW) infants, are known to have higher nutritional requirements than term infants [1,2]. For all infants, the mother’s own milk is considered the best first feeding choice, as recommended by the World Health Organization, by the European Society for Pediatric Gastroenterology Hepatology and Nutrition, and by the American Academy of Pediatrics [3]. Human milk contains a variety of bioactive compounds that are involved in the shaping and modulation of the gastrointestinal tract and immune system, as well as in brain development, in addition to its nutrient composition [3,4].

An appropriate nutrition constitutes a prominent factor for a good quality of life during childhood and adulthood. Several disorders are due to nutrient deficiency in the first months of life. For preterm infants, low sodium level may lead to hyponatraemia, and calcium and phosphorus intake could be below the intake needed to achieve foetal rates of bone mineral accretion [1,2,5,6,7]. For preterm and LBW infants, an adequate protein intake is crucial to limit post-natal growth deficit; after the first weeks, the protein content of human milk is too low to ensure adequate growth. Considering that the growth deficit is difficult to recover during the unstable phase, the feeding of clinically stable preterm infants becomes crucial to recuperate the accumulated deficit, and to prevent morbidities related to postnatal growth failure. Therefore, supplementation of human milk (HM) with more than one nutritional component is a common practice in most neonatal intensive care units (NICUs) [3].

Since mothers’ own milk has been acknowledged as the gold standard in infant nutrition, industrial strategies have been directed toward mimicking the nutritional profile of human breast milk for optimizing infant growth and development. Protein-based fortifiers for human milk and preterm infant formulas (PIF) are being continuously improved by manufacturers, to cope with new nutritional trends and recommendations for adequate preterm growth. The source and/or the quality of both the protein and lipid fractions of preterm infant formulas and supplements may have important consequences on tolerability, palatability, allergenicity and nutritional outcomes. For protein-based fortifiers, bovine milk constituents (caseins, whey proteins and hydrolysates thereof) are the most common sources of nitrogen. Plant oils (such as palm, sunflower and coconut) and fish oils are usually added as a fat source [8,9]. Carbohydrate sources are mostly represented by lactose and maltodextrins (corn syrup solids). In addition, a variety of vitamins, mineral mixes, and other ingredients are added, depending on the manufacturer, although beneficial claims associated with some ingredients are often largely to be demonstrated [10]. Besides macro and micronutrients, the presence of bioactive compounds with possible health benefits, as well as that of contaminants deriving from food alteration, should be carefully evaluated. To ensure microbiological safety and long shelf life, preterm infant formulas usually undergo extensive heat treatment at high temperatures. For this reason, and due to their high concentration of nutrients, preterm infant formulas and fortifiers are more prone to thermally induced degradation reactions than standard milk products. In particular, lipid and protein modifications, occurring as a consequence of product manufacturing, may adversely affect the quality of food products intended for preterm infants, and may increase the intake in oxidative and oxidized compounds [11,12,13]. An increase in antioxidant supply, as well as a reduced oxidized species intake, is specifically relevant for preterm infants, which have an immature antioxidant defence system and are frequently exposed to oxidative stress caused by infection, mechanical ventilation, intravenous nutrition, and blood transfusions [14,15]. Previous studies have reported the quantification of biomarkers derived from oxidation processes, such as malondialdehyde (MDA), isoprostanes and protein carbonyls, as a reliable strategy for the evaluation of in vivo oxidative stress in preterm newborn [16,17,18].

In recent years, innovative efforts have been made to provide the best compromise between the nutritional needs of preterm infants and the maximum intake in bioactive compounds. Fortifiers based on human milk proteins [19,20] and on donkey milk [21,22] have been studied as an alternative to bovine milk-based fortifiers, and, in the case of human milk-based fortifiers, commercialized. A HM based fortifier has been demonstrated to decrease the duration of parenteral nutrition and to reduce rates of necrotizing enterocolitis [19,20]. In a recently published clinical trial, a new donkey milk-derived fortifier brought about a reduction in feeding intolerance, bilious gastric residuals and vomiting in hospitalized preterm and LBW infants [21]. In all the aforementioned studies, the use of breast milk was recognized as the best nutritional strategy in preterm newborn nutrition.

The aim of this work is to compare the biochemical composition of 9 products for preterm infant nutrition (human milk, preterm infant formulas (PIFs), multicomponent fortifiers and protein concentrates), with specific focus on in vitro antioxidant activity and content in oxidative compounds.

## 2. Materials and Methods

### 2.1. Bovine Milk-Based Infant Formulas and Human Milk Fortifiers

Four commercially available bovine milk-based products for preterm infant nutrition—two PIFs (Prenidina and Plasmon 0) and two multicomponent fortifiers for human milk (FM85 and Milte) and one protein concentrate (Protifar)—were considered. Liquid products were lyophilized before analysis, and stored at −20 °C.

### 2.2. Donkey Milk-Based Human Milk Fortifiers

Two experimental donkey milk (DM)-based fortifiers were produced on a pilot scale. The two experimental DM-based human milk fortifiers were produced by ultrafiltration of pasteurized donkey milk. The ultrafiltration process was designed to obtain two different fortifiers, characterized by two protein concentration levels, comparable to the corresponding bovine milk derived commercial products (FM85 and Milte). Protein concentrates were pasteurized, aseptically lyophilized and packed, and analysed to ensure compliance to microbiological criteria for infant food products. The two experimental DM–based products were used in a clinical trial, as previously described [21,22].

### 2.3. Raw and Pasteurized Human Milk

Both raw and pasteurized HM samples were obtained from the human milk bank (HMB) of the Città della Scienza e della Salute of Torino, Italy, from 4 healthy donor mothers. All mothers were enrolled as donors by the bank, and donations were performed in accordance with the HMB guidelines. An ethical review process was not required for this study. At first donation, donors signed a written informed consent, and were informed that only milk samples stored in excess of the needs of their infants would have been used for research purposes. The milk specimens from term mothers were collected in sterile bisphenol-free polypropylene bottles using a breast pump and stored, by the HMB, at −20 °C until processed. The HM samples were thawed overnight in a refrigerator, and pooled before pasteurization and lyophilisation. Half of the pooled sample was pasteurized in the HMB facility at 62.5 °C for 30 min (holder pasteurization) before lyophilisation. The raw (RHM) and pasteurized samples (DHM) were finally lyophilized, to increase the stability of samples, without modifying the nutritional properties, the content of oxidised species and enzymatic activities of human milk [23,24].

### 2.4. Chemical Composition Determination for Experimental Products and Human Milk Samples

The chemical composition of experimental products and human milk samples was determined in terms of gross energy, lactose, total protein and fat content. Gross energy was determined in excess oxygen by adiabatic bomb calorimeter (Mod. 700, IKA GmbH & Co., Staufen, Germany), using benzoic acid as a reference (26.454 MJ/kg). Protein concentration was determined by UV spectroscopy at 280 nm using bovine serum albumin as standard, and by the Dumas method using a RapidN III device (Elementar Analysensysteme GmbH e Isoprime Ltd, Lomazzo, Co, Italy). The total fat content was measured gravimetrically on ether extract after acid hydrolysis according to the ISTISAN method A [25]. Lactose content was measured by enzymatic assay kit (R-Biopharm AG, Darmstadt, Germany).

### 2.5. Trolox Equivalent Antioxidant Capacity (TEAC)

The Trolox (6-hydroxy-2,5,7,8-tetramethylchroman-2-carboxylic acid) equivalent antioxidant capacity (TEAC) of aqueous suspensions from preterm foods and supplements (50 mg/mL, and dilutions thereof, where necessary) was determined using the 2,2′-azino-bis(3-ethylbenzothiazoline-6-sulphonic acid) (ABTS) radical cation decolorization assay [26]. ABTS was activated in a 2.45 mmol/L solution of potassium persulfate for 16 h. The stock solution was diluted with water up to a final absorbance of 0.70 ± 0.02 at λ = 734 nm. The ABTS^•+^ solution was pipetted in 2 mL doses to test tubes placed in a block heater (TH-24, Meditherm, Poland) warmed to 30 °C, and, after temperature settling, 0.02 mL of tested suspensions were added. The absorbance was recorded at λ = 734 nm (DU-7500 spectrophotometer, Beckman, Brea, CA, USA) after centrifugation for 5 min at 4000× *g* (MiniSpin plus, Eppendrof, Hamburg, Germany). The results were calculated using a curve for Trolox as reference, and expressed as µmol Trolox equivalents per 100 mL (for liquid foods) or per 1 g of proteins (for protein fortifiers). For each milk sample type, fortifier and concentrate, three technical replicates were analysed.

### 2.6. Antioxidant Activity Against DPPH

Antiradical activity of aqueous resuspensions (50 mg/mL) against DPPH^•^ (2,2-diphenyl-1-picrylhydrazyl radical) was measured according to the method described by Brand-Williams et al. [27]. The reaction was run by immediately vortexing 0.1 mL from each tested resuspension with 0.25 mL of 1 mmol/L methanolic solution of DPPH and 2 mL of methanol. After 20 min, the reaction mixtures were centrifuged for 5 min at 4000× *g* (MiniSpin plus, Eppendrof, Hamburg, Germany) and absorbance was read at λ = 517 nm (DU-7500Beckman). Radical scavenging activity was expressed as the inhibition percentage of free radicals by the sample and was calculated as [1 − (test sample absorbance/DPPH^•^ solution absorbance)] × 100 [28], normalized over the different protein content for protein fortifiers and expressed on a 100 mL volume for liquid foods. For each milk sample type, fortifier and concentrate, three technical replicates were analysed.

### 2.7. Measurement of Oxidative Content (Protein Carbonyls and Malondialdehyde)

The protein carbonyl (PC) content was determined using the methodology adapted from Fenaille et al. [11] as previously reported [29]. An aliquot of aqueous resuspensions (10 mg/mL) was incubated with 10 mmol/L of 2,4-dinitrophenylhydrazine (DNPH) in 2 mol/L HCl (1 mL volume), for 30 min at room temperature. Proteins were then precipitated by adding 10% (*w*/*v*, final concentration) trichloroacetic acid, and recovered by centrifugation for 5 min at 7500× *g* (Micromax RF centrifuge, International Equipment Company, Needham, MA). Protein pellets were washed 3 times with 1 mL of ethanol/ethyl acetate 50:50 (*v*/*v*) to remove free DNPH reagent, and redissolved in 1 mL of 6 mol/L guanidine hydrochloride, pH 2.3. Protein carbonyls were determined by UV spectrophotometry at λ = 370 nm, using an extinction coefficient of 2.2 × 10^4^ L mol^−1^·cm^−1^, with a Mini Spec UV-vis spectrophotometer (Shimadzu, Kyoto, Japan). Results were reported as µmol of PC per 100 mL (for liquid foods) or per 1 g of proteins (for protein fortifiers). The content of MDA of preterm foods and supplements was analysed according to Seljeskog et al. [30], with some adaptations. Extracted samples were injected in the HPLC system consisted of a Dionex P680 pump (Dionex, Sunnyvale, CA, USA), a RF-2000 fluorimetric detector (λ_ex_ = 525, λ_em_ = 560), a thermostated column compartment TCC-100, a ASI100 autosampler series and a Chromeleon® 6 data handling system (Dionex). The analytical column was a Gemini LC-18 column (150 × 4.6 mm, 5 µm particles) (Phenomenex, Torrance, CA, USA) preceded by an Analytical Guard Cartridge System (Phenomenex). The system ran isocratically with a mobile phase containing 50 mmol/L KH_2_PO_4_-methanol-acetonitrile (72:17:11, *v*/*v*/*v*), at a flow rate of 0.8 mL/min. A standard curve was made from 1,1,3,3-tetraethoxypropane (TEP) dissolved in methanol and diluted at concentrations of 10.0, 5.0, 2.5, 1.25, 0.62 and 0.21 μmol/L. Results were expressed as nmol MDA per 100 mL (for liquid foods) or per 1 g of proteins (for protein fortifiers). For each milk sample type, fortifier and concentrate, three technical replicates were analysed.

### 2.8. In Vitro Antioxidant Activity

Preparation of erythrocytes. According to the regulations of “Fondazione G. Monasterio CNR-Regione Toscana”, human blood samples were collected from healthy blood donors upon informed consent, for the use of residual blood for research purposes. Samples of human blood from three healthy volunteers were collected in ethylenediaminetetracetic acid (EDTA)-treated tubes, and centrifuged (Jouan CR3i, Thermo Electron Corporation, UK), for 10 min at 2300× *g* at 4 °C. Plasma and buffy coat were discarded, and erythrocytes were washed twice with PBS at pH 7.4. 

Cellular antioxidant activity assay in red blood cells (CAA-RBC). The antioxidant activity of preterm foods and supplements was evaluated in an in vitro system with red blood cells (RBC) [31]. Erythrocytes were diluted 1:100 (*w*/*v*) in PBS at pH 7.4 and incubated for 1 h at 37 °C with 15 µmol/L of 2′,7′-dichlorodihydrofluorescein diacetate (DCFH-DA) and PBS (control) or tested samples (100 g/L water dissolved and diluted with PBS). At the end of the incubation, RBC were washed twice, resuspended in cold PBS and transferred to a 96-well microplate. After that, 1.2 mM 2,2′-azobis(2-methylpropionamidine) dihydrochloride (AAPH) was added to the cell suspension and the fluorescence was read each 8 seconds for 12 cycles at λ_ex_ = 485 nm and λ_em_ = 535 nm by Victor^TM^ 3 Multilabel Plate Reader (Waltham, MA) in order to generate a curve. Each value was expressed as CAA units, according to the Wolfe and Liu formula [32]:

CAA unit = 100 − (∫SA/∫CA) × 100

where ∫SA is the integrated area of the sample curve and ∫CA is the integrated area of the control curve.

Hemolysis test. Hemolysis was measured according to the method of Mikstacka et al. [33] using AAPH, a generator of peroxyl radicals, to induce RBC lysis. Briefly, 450 μL of a 5% (*w*/*v*) erythrocyte suspension in PBS was pre-incubated with tested resuspensions of preterm foods and supplements (100 g/L water) at 37 °C for 1 h, then exposed to 50 μL of 50 mmol/L AAPH at 37 °C for 4 h. The samples were centrifuged (Jouan CR3i) for 5 min at 1000× *g*, and the absorbance was read at λ = 540 nm. Control and blank samples were represented by erythrocytes pre-incubated only with AAPH or with PBS (blank). The values reported are the percentage of hemolysis compared to the control.

### 2.9. Statistical Analysis

In order to allow direct comparison among the different products under assay, data were expressed on a 100 mL volume basis of human milk (both raw and pasteurized) and reconstituted PIFs, while data from preterm fortifiers (both multicomponent and concentrate) where expressed per gram of protein. The statistical analyses of products expressed on volume basis and of those expressed per gram of protein were performed separately. Before analysis, data were standardized by subtracting mean values and dividing by standard deviations of each parameter, and subjected to one-way and multivariate analysis of variance and to hierarchical clustering. One-way ANOVA was performed by F-test followed by Tukey’s post-hoc, or by the Kruskal-Wallis test followed by Dunn’s post-hoc for, respectively, non-significant and significant Levene’s test for homogeneity of variances. Multivariate ANOVA and hierarchical clustering (UPGMA algorithm of Manhattan distances) were performed by analyzing all standardized values. All statistical analyses were performed by the PAST 3 software package [34].

## 3. Results and Discussion

The infant foods and human milk fortifiers considered in the present research were selected to be representative of different sources of nutrients, on the basis of the manufacturers’ labels (Table 1), with a focus on the protein source and status (whole or hydrolysed). In particular, we selected two PIFs, both containing bovine milk proteins, although differing in terms of the protein type. PIF1 contained whole caseins and whey proteins, with added lactoferrin, while PIF2 contained partially hydrolysed whey proteins. Both PIFs contained added docosahexaenoic acid (DHA), arachidonic acid (ARA) and medium-chain triacylglycerols (MCT), plant oils, maltodextrins and lactose. PIF1 also contained GOS as prebiotics. In addition, a human breast milk sample before (RHM) and after pasteurization (DHM) was considered. 

Of the two considered bovine milk-based fortifiers (BM1 and BM2), BM1 contained extensively hydrolysed whey proteins and maltodextrins, while BM2 contained whole whey proteins, added lactoferrin, lutein and prebiotics (GOS and FOS). We also considered two experimental products derived from whole donkey milk, without added ingredients. A protein concentrate (85% protein) derived from skimmed bovine milk (BC) was also sampled, containing whole caseins and whey proteins. The nutrient composition and the concentrations of measured oxidative status parameters were expressed as per gram of protein for the fortifiers and the protein concentrate, since the neonatal intensive care unit guidelines and the clinical practice indicate preterm infant requirements as g of added protein per 1 kg of body weight per day. For human milk and reconstituted PIFs, the aforementioned parameters were expressed on a 100 mL volume basis, since the starting of fortification is advisable when enteral intake reaches 100 mL/kg/day [4].

**Figure 1:**
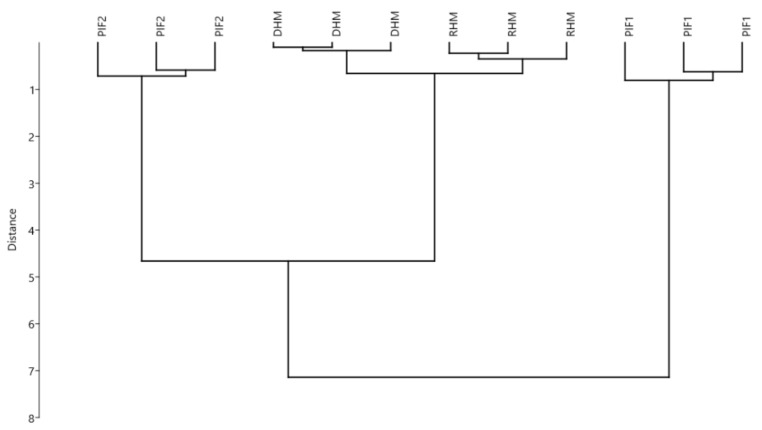
Multivariate cluster analysis (UPGMA algorithm – Manhattan distance) of different preterm infant formula milks (per 100 mL product). Variables (*n* = 3): antioxidant capacities (measured as TEAC and DPPH), protein carbonyl and malondialdehyde contents. RHM: raw human milk; DHM: holder pasteurized human milk; PIF1: preterm infant formula type 1; PIF2: preterm infant formula type 2.

The analyses performed included 3 assay types aimed at determining the oxidative load of the samples by measuring: (i) the antioxidant activity/capacity, by measuring radical scavenging (TEAC, DPPH), (ii) the antioxidant activity/capacity, by using erythrocytes as indicators (Haemolysis, CAA-RBC),and (iii) quantification of intermediate/final oxidation products (MDA, PC). An overview of the differences between the samples was first performed by a multivariate approach, including all oxidative status parameters. Figure 1 reports the results of multivariate hierarchical clustering of samples PIFs, RHM and DHM. The clusters showed a higher similarity between PIF2 and human milk samples, with respect to PIF1, in term of oxidation status. Multivariate ANOVA revealed that the observed differences in the oxidative load (as measured by considering all the parameters together) were significant (*p* < 0.001).

In order to gain a deeper insight, a one-way analysis of variance on the single parameters was performed. Results are reported in Table 2. In accordance with clustering results in Figure 1, human milk samples showed a similar oxidative status, although antioxidant activity as measured by TEAC was higher for pasteurized HM (DHM). In a previous study, Martysiak-Żurowska et al. [35] had found no significant difference (*p* ≥ 0.05) in TEAC between DHM and RHM. In turn, a decreased antioxidant content (carotenoids and tocopherols) has been reported for donor milk with respect to fresh breast milk by other authors [36]. As for human milk substitutes, both PIFs showed a higher content of oxidized compounds, with respect to human milk, in particular for MDA content.

The formula containing partially hydrolysed proteins (PIF2) showed a significantly lower content in protein carbonyls than the formula containing whole bovine proteins (PIF1). This may be due to the specific PIF2 protein profile, which is represented by 100% partially hydrolysed whey proteins. Nevertheless, as hydrolysed proteins may escape precipitation by TCA, which is required in sample preparation for PC analysis, we cannot exclude that the carbonyl content of PIF2 is underestimated. PIF1 showed a lower antioxidant capacity with respect to human milk, both measured as TEAC and DPPH, while PIF2 showed a higher TEAC than human milk, probably due to its peculiar peptide composition. It is known that even limited hydrolysis of milk proteins can significantly increase the TEAC of hydrolysates, as compared to parent proteins [37]. Encrypted peptides are able to interact with radical species or to inhibit oxidative reactions thanks the greater exposure of antioxidant amino acids compared to whole proteins [38]. However, it is worth mentioning that, besides protein and peptides, other compounds such as vitamin E, carotenoids and flavonoids could contribute to antioxidant activity in infant formulas [39]. Taking together these characteristics, feeding with PIF2 rather than PIF1 would seem to result in a lower oxidative load for the preterm newborn, although the best choice is always human milk, irrespective of pasteurization. These data confirm those found by other authors by in vivo studies [40,41,42,43,44], who reported higher excretion of oxidised compounds in infants consuming infant formulas, with respect to human milk.

**Figure 2:**
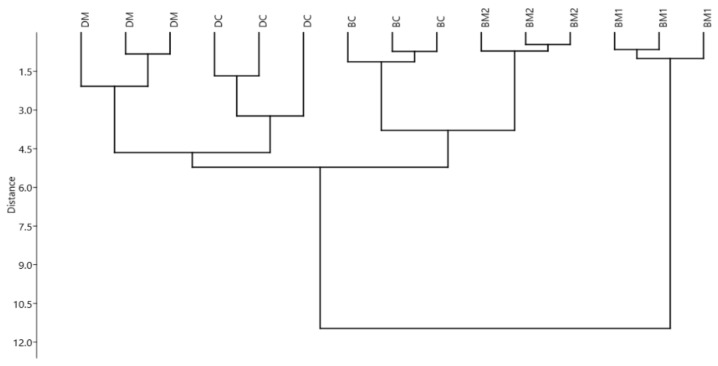
Multivariate cluster analysis (UPGMA algorithm – Manhattan distance) of different human milk fortifiers and protein concentrate (per g of added protein). Variables (*n* = 3): antioxidant capacities (measured as TEAC, DPPH, and CAA (Cellular antioxidant activity)), haemolysis, protein carbonyl and malondialdehyde contents. BM1: bovine milk preterm fortifier type 1; BM2: bovine milk preterm fortifier type 2; DM: donkey milk preterm fortifier; DC: donkey milk preterm concentrate; BC: bovine milk concentrate.

Figure 2 reports the results of multivariate hierarchical clustering of human milk fortifiers and protein concentrate, according to their oxidative status. In this case, three clusters were observed: one represented by DM derived products, one represented by BM based products containing non-hydrolysed proteins, and the last one represented by BM1, which contains highly hydrolysed bovine whey proteins.

Also in this case, MANOVA revealed a significant difference between the samples (*p* < 0.001). In accordance with clustering results, BM1 was significantly different from the other fortifiers for most parameters, and, in particular, it showed a better antioxidant capacity, probably due to the protein content type (100% extensively hydrolysed whey proteins), as already seen for PIF2 (Table 3). Peptides released from whey proteins during hydrolysis are known to have strong antioxidant activities [45]. Nevertheless, also in this case, the amount of oxidized protein compounds could be underestimated, due to the lack of precipitation for small peptides. Accordingly, the amount of MDA was significantly higher for BM1 than for the non-hydrolysed fortifiers. BM-derived fortifiers (with the exception of BM1) seemed to have lower antioxidant capacities, but also lower MDA and PC contents than donkey milk-derived fortifiers (Table 3). This may be partially due to the fact that the formation of oxidation products, such as PC and MDA, is highly dependent on the amount of reducing sugars and on lipids, which are triggering substrates for oxidation. Both lactose content and lipid content are higher in donkey milk-derived products, accordingly.

One limitation of the present study is that samples were technical replicates of the same batches. In order to generalize our findings, further batches will need to be analysed.

In general, results suggest that, with the exception of BM1, fortifying human milk result in an increase in the oxidative load. Although few studies are available on the issue, Friel et al. [42] pointed out an elevated urinary excretion of F2-Isoprostenes (an in vivo marker of oxidative stress) for preterm infants who had their mother’s milk fortified with bovine-milk based fortifiers, and, recently, the same group confirmed this observation also for infants receiving fortifiers produced by concentrated human milk [46]. Human milk-based fortifiers [19,20,47] were not included in the present study, since they are currently not marketed in Europe. The increased excretion of F2-isoprostanes in preterm infants receiving fortified human milk may also be due to an increased imbalance of antioxidant defences, resulting from the higher protein and macronutrient intake in these infants.

## 4. Conclusions

The preterm infant formulas showed higher oxidative loads when compared to human milk. This is especially true as far as MDA is concerned, since the addition of fortifiers to human milk may result in higher MDA content. On the other end, the addition of fortifiers to human milk may increase the antioxidant capacity, although further confirmations are necessary. Among bovine milk proteins, hydrolysed whey proteins provided a higher antioxidant capacity than whole proteins. These results could be a basis for improving the quality of foods and supplements intended for the nutrition of preterm infants.

## Figures and Tables

**Table 1:** Composition of commercial and experimental products used in the current study (source: manufacturers; “Latti formulati in commercio in Italia” by De Curtis M. and Gasparrini E.).

		Energy	Protein				Lipid		Carbohydrate					
		100 mL										1 g protein		
**Preterm formulas**	**Name**	**Kcal**	**Content (g)**	**W/C**	**Hydr**	**Source**	**Content (g)**	**Source**	**Content (g)**	**Type**	**Added ingredient**	**Energy**	**Lipid**	**Carb**
	PIF1	79	2.4	60–40	-	Bovine	3.9	Plant	8.7	Maltodextrins lactose	Lactoferrin/ DHA-ARA-MCT/ GOS	32.9	1.63	3.6
	PIF2	81	2.9	100–0	+	Bovine	4.0	Plant	8.4	Maltodextrins lactose	DHA-ARA-MCT	27.9	1.38	2.9
		100 g										1 g protein		
**Fortifiers and concentrates**	**Name**	**Kcal**	**Content (g)**	**W/C**	**Hydr**	**Source**	**Content (g)**	**Source**	**Content (g)**	**Type**	**Added ingredient**	**Energy**	**Lipid**	**Carb**
	BM1	347	20	100–0	+++	Bovine	0.4	?	66	Maltodextrins		17.4	0.02	3.3
	BM2	260	35	100–0	-	Bovine	0.4	Soybean	38	Maltodextrin	Lactoferrin/	7.4	0.01	1.1
											GOS FOS/			
											Lutein			
	BC	368	87	20–80	-	Bovine	1.6	Bovine	1.2	Lactose		4.2	0.02	0.0
	DM	390	22	60–40	-	Donkey	3.6	Donkey	59	Lactose		17.7	0.16	2.7
	DC	418	43	60–40	-	Donkey	6.1	Donkey	33	Lactose		9.7	0.14	0.8

W/C: whey/casein ratio; Hydr:—whole; + partially hydrolysed; +++ extensively hydrolysed protein; GOS: galactooligosaccharides; FOS: fructooligosaccharides; DHA: docosahexaenoic acid; ARA: arachidonic acid; MCT: medium-chain triacylglycerols; PIF: preterm infant formula; BM: bovine milk-based fortifier; BC: bovine concentrate; DM: donkey milk-based fortifier; DC: donkey concentrate.

**Table 2:** Selected oxidative status indicators (means ± standard deviations; *n* = 3) for different preterm foods (100 mL).

	TEAC ^†^		DPPH ^†^		PC ^‡^		MDA ^†^	
**RHM**	61.3 ± 2.2	*C*	10.8 ± 0.7	*A*	0.6 ± 0.1	*A*	195 ± 5	*A*
**DHM**	82.0 ± 1.7	*B*	10.6 ± 0.2	*A*	0.9 ± 0.1	*A*	199 ± 6	*A*
**PIF1**	10.2 ± 0.2	*D*	2.2 ± 0.3	*B*	14.3 ± 2.2	*B*	1329 ± 72	*C*
**PIF2**	131.7 ± 3.4	*A*	2.7 ± 0.5	*B*	1.8 ± 0.2	*A*	867 ± 191	*B*

TEAC: antioxidant capacity measured as Trolox Equivalents (µmol); DPPH: antioxidant capacity measured as % inhibition of DPPH radicals; PC: protein carbonyls (µmol); MDA: malondialdehyde (nmol). RHM: raw human milk; DHM: holder pasteurized human milk; PIF1: preterm infant formula type 1; PIF2: preterm infant formula type 2. Significantly different samples are indicated by different italicized letter (A, B, C and D) following the Tukey (†) or Dunn’s (‡) post-hoc test (*p* < 0.05).

**Table 3:** Selected oxidative status indicators (means ± standard deviations; *n* = 3) for different human milk fortifiers or protein concentrates (per g of added protein).

	TEAC ^†^		DPPH ^†^		Haemolisis ^‡^		CAA ^†^		PC ^‡^		MDA ^†^	
**BM1**	470 ± 4	*A*	9.6 ± 0.1	*A*	11 ± 1	*A*	83 ± 3	*A*	0.13 ± 0.05	*A*	95 ± 12	*A*
**BM2**	37 ± 0	*E*	<0.3		29 ± 1	*AB*	41 ± 3	*B*	0.56 ± 0.08	*AB*	14 ± 2	*C*
**BC**	141 ± 2	*C*	0.3 ± 0.1	*B*	62 ± 6	*B*	19 ± 1	*D*	0.55 ± 0.13	*AB*	10 ± 2	*C*
**DM**	113 ± 2	*D*	<0.3		30 ± 4	*AB*	33 ± 3	*C*	0.81 ± 0.18	*B*	92 ± 7	*A*
**DC**	193 ± 2	*B*	<0.3		54 ± 20	*B*	24 ± 1	*D*	1.02 ± 0.24	*B*	60 ± 11	*B*

TEAC: antioxidant capacity measured as Trolox Equivalents (µmol); DPPH: antioxidant capacity measured as % inhibition of DPPH radicals; PC: protein carbonyls (µmol); MDA: malondialdehyde (nmol); Haemolysis: % red blood globules haemolysed with respect to AAPH (100%); CAA: antioxidant capacity (CAA units). BM1: bovine milk preterm fortifier type 1; BM2: bovine milk preterm fortifier type 2; DM: donkey milk preterm fortifier; DC: donkey milk preterm concentrate; BC: bovine milk concentrate. Significantly different samples are indicated by different italicized letter (A, B, C, D and E) following Tukey (†) or Dunn’s (‡) post-hoc test (*p* < 0.05).

## Fundings

Part of this research was funded by the “Short Term Mobility 2015” grant program of Consiglio Nazionale delle Ricerche (CNR) to L.P.

## References

[1] Carlson S.J., Ziegler E.E. (1998). Nutrient intake and growth of very low birth weight infants. J. Perinatol..

[2] De Curtis M., Rigo J. (2012). The nutrition of preterm infants. Early Hum. Dev..

[3] Arslanoglu S., Boquien C.-Y., King C., Lamireau D., Tonetto P., Barnett D., Bertino E., Gaya A., Gebauer C., Grovslien A. (2019). Fortification of human milk for preterm infants: Update and recommendations of the European Milk Bank Association (EMBA) Working Group on human milk fortification. Front. Pediatr..

[4] Dutta S., Singh B., Chessell L., Wilson J., Janes M., McDonald K., Shahid S., Gardner V.A., Hjartarson A., Purcha M. (2015). Guidelines for feeding very low birthweight infants. Nutrients.

[5] Embleton N.E., Pang N., Cooke R.J. (2001). Postnatal malnutrition and growth retardation: An inevitable consequence of current recommendations in preterm infants?. Pediatrics.

[6] Heird W.C. (2001). Determination of nutritional requirements in preterm infants, with special reference to ‘catch-up’ growth. Semin. Neonatol..

[7] Horbar J.D., Ehrenkranz R.A., Badger G.J., Edwards E.M., Morrow K.A., Soll R.F., Buzas J.S., Bertino E., Gagliardi L., Bellu R. (2015). Weight growth velocity and postnatal growth failure in infants 501 to 1500 grams: 2000–2013. Pediatrics.

[8] Królczyk J.B., Dawidziuk T., Janiszewska-Turak E., Sołowiej B. (2016). Use of whey and whey preparations in the food industry—A review. Polish J. Food Nutr. Sci..

[9] Lapillonne A., Groh-Wargo S., Lozano Gonzalez C.H. (2013). Lipid needs of preterm infants: Updated recommendations. J. Pediatr..

[10] Koo W., Tice H. (2018). Human milk fortifiers do not meet the current recommendation for nutrients in very low birth weight infants. J. Parenter. Enter. Nutr..

[11] Fenaille F., Parisod V., Visani P., Populaire S., Tabet J.-C., Guy P.A. (2006). Modifications of milk constituents during processing: A preliminary benchmarking study. Int. Dairy J..

[12] Pischetsrieder M., Henle T. (2012). Glycation products in infant formulas: Chemical, analytical and physiological aspects. Amino Acids.

[13] Scheidegger D., Radici P.M., Vergara-Roig V.A., Bosio N.S., Pesce S.F., Pecora R.P., Romano J.C.P., Kivatinitz S.C. (2013). Evaluation of milk powder quality by protein oxidative modifications. J. Dairy Sci..

[14] Inayat M., Bany-Mohammed F., Valencia A., Tay C., Jacinto J., Aranda J.V., Beharry K.D. (2015). Antioxidants and biomarkers of oxidative stress in preterm infants with symptomatic patent ductus arteriosus. Am. J. Perinatol..

[15] Moore T.A., Ahmad I.M., Zimmerman M.C. (2018). Oxidative stress and preterm birth: An integrative review. Biol. Res. Nurs..

[16] Cháfer-Pericás C., Rahkonen L., Sánchez-Illana A., Kuligowski J., Torres-Cuevas I., Cernada M., Cubells E., Nuñez-Ramiro A., Andersson S., Vento M. (2015). Ultra high performance liquid chromatography coupled to tandem mass spectrometry determination of lipid peroxidation biomarkers in newborn serum samples. Anal. Chim. Acta.

[17] Hsiao C.C., Chang J.C., Tsao L.Y., Yang R.C., Chen H.N., Lee C.H., Lin C.Y., Tsai Y.G. (2017). Correlates of elevated interleukin-6 and 8-hydroxy-2′-deoxyguanosine levels in tracheal aspirates from very low birth weight infants who develop bronchopulmonary dysplasia. Pediatr. Neonatol..

[18] Slater L., Asmerom Y., Boskovic D.S., Bahjri K., Plank M.S., Angeles K.R., Phillips R., Deming D., Ashwal S., Hougland K. (2012). Procedural pain and oxidative stress in premature neonates. J. Pain.

[19] Sandhu A., Fast S., Bonnar K., Baier R.J., Narvey M. (2017). Human-based human milk fortifier as rescue therapy in very low birth weight infants demonstrating intolerance to bovine-based human milk fortifier. Breastfeed. Med..

[20] Sullivan S., Schanler R.J., Kim J.H., Patel A.L., Trawöger R., Kiechl-Kohlendorfer U., Chan G.M., Blanco C.L., Abrams S., Cotten C.M. (2010). An Exclusively human milk-based diet is associated with a lower rate of necrotizing enterocolitis than a diet of human milk and bovine milk-based products. J. Pediatr..

[21] Bertino E., Cavallarin L., Cresi F., Tonetto P., Peila C., Ansaldi G., Raia M., Varalda A., Giribaldi M., Conti A. (2019). A Novel donkey milk-derived human milk fortifier in feeding preterm infants: A randomized controlled trial. J. Pediatr. Gastroenterol. Nutr..

[22] Coscia A., Bertino E., Tonetto P., Peila C., Cresi F., Arslanoglu S., Moro G.E., Spada E., Milani S., Giribaldi M. (2018). Nutritional adequacy of a novel human milk fortifier from donkey milk in feeding preterm infants: Study protocol of a randomized controlled clinical trial. Nutr. J..

[23] Martysiak-Żurowska D., Puta M., Rodzik A., Malinowska-Pańczyk E. (2017). The effect of lyophilization on selected biologically active components (vitamin c, catalase, lysozyme), total antioxidant capacity and lipid oxidation in human milk. Żywność. Nauk. Technol. Jakość.

[24] Salcedo J., Gormaz M., López-Mendoza M.C., Nogarotto E., Silvestre D. (2015). Human milk bactericidal properties: Effect of lyophilization and relation to maternal factors and milk components. J. Pediatr. Gastroenterol. Nutr..

[25] Baldini M., Fabietti F., Giammarioli S., Onori R., Orefice L., Stacchini A. (1996). Analytical methods used in food chemical control. Rapp. ISTISAN.

[26] Re R., Pellegrini N., Proteggente A., Pannala A., Yang M., Rice-Evans C. (1999). Antioxidant activity applying an improved ABTS radical cation decolorization assay. Free Radic. Biol. Med..

[27] Brand-Williams W., Cuvelier M.E., Berset C. (1995). Use of a free radical method to evaluate antioxidant activity. LWT Food Sci. Technol..

[28] Barros L., Ferreira M.-J., Queirós B., Ferreira I.C.F.R., Baptista P. (2007). Total phenols, ascorbic acid, β-carotene and lycopene in Portuguese wild edible mushrooms and their antioxidant activities. Food Chem..

[29] Levine R.L., Garland D., Oliver C.N., Amici A., Climent I., Lenz A.-G., Ahn B.-W., Shaltiel S., Stadtman E.R. (1990). Determination of carbonyl content in oxidatively modified proteins. Methods Enzymol..

[30] Seljeskog E., Hervig T., Mansoor M.A. (2006). A novel HPLC method for the measurement of thiobarbituric acid reactive substances (TBARS). A comparison with a commercially available kit. Clin. Biochem..

[31] Frassinetti S., Gabriele M., Caltavuturo L., Longo V., Pucci L. (2015). Antimutagenic and antioxidant activity of a selected lectin-free common bean (Phaseolus vulgaris L.) in two cell-based models. Plant Foods Hum. Nutr..

[32] Wolf K.L., Liu R.H. (2007). Cellular Antioxidant Activity (CAA) Assay for Assessing Antioxidants, Foods, and Dietary Supplements. J. Agric. Food Chem..

[33] Mikstacka R., Rimando A.M., Ignatowicz E. (2010). Antioxidant effect of trans-resveratrol, pterostilbene, quercetin and their combinations in human erythrocytes in vitro. Plant Foods Hum. Nutr..

[34] Hammer Ø., Harper D.A.T., Ryan P.D. (2001). PAST: Paleontological statistics software package for education and data analysis. Palaeontol. Electron..

[35] Martysiak-Zurowska D., Puta M., Barczak N., Dabrowska J., Malinowska-Pańczyk E., Kiełbratowska B., Kołodziejska I. (2017). Effect of high pressure and sub-zero temperature on total antioxidant capacity and the content of vitamin C, fatty acids and secondary products of lipid oxidation in human milk. Polish J. Food Nutr. Sci..

[36] Hanson C., Lyden E., Furtado J., Van Ormer M., Anderson-Berry A. (2016). A comparison of nutritional antioxidant content in breast milk, donor milk, and infant formulas. Nutrients.

[37] Dryáková A., Pihlanto A., Marnila P., Čurda L., Korhonen H.J.T. (2010). Antioxidant properties of whey protein hydrolysates as measured by three methods. Eur. Food Res. Technol..

[38] Lamothe S., Guérette C., Dion F., Sabik H., Britten M. (2019). Antioxidant activity of milk and polyphenol-rich beverages during simulated gastrointestinal digestion of linseed oil emulsions. Food Res. Int..

[39] Lindmark-Månsson H., Åkesson B. (2000). Antioxidative factors in milk. Br. J. Nutr..

[40] Aycicek A., Erel O., Kocyigit A., Selek S., Demirkol M.R. (2006). Breast milk provides better antioxidant power than does formula. Nutrition.

[41] Chen Y., Fantuzzi G., Schoeny M., Meier P., Patel A.L. (2019). High-dose human milk feedings decrease oxidative stress in premature infant. J. Parenter. Enter. Nutr..

[42] Friel J.K., Diehl-Jones B., Cockell K.A., Chiu A., Rabanni R., Davies S.S., Jackson Roberts L. (2011). Evidence of oxidative stress in relation to feeding type during early life in premature infants. Pediatr. Res..

[43] Friel J.K., Martin S.M., Langdon M., Herzberg G.R., Buettner G.R. (2002). Milk from mothers of both premature and full-term infants provides better antioxidant protection than does infant formula. Pediatr. Res..

[44] Ledo A., Escrig R., Brugada M., Aguar M., Saenz P., Vento M., Arduini A., Asensi M.A., Sastre J. (2009). Human milk enhances antioxidant defenses against hydroxyl radical aggression in preterm infants. Am. J. Clin. Nutr..

[45] Mann B., Athira S., Sharma R., Kumar R., Sarkar P. (2019). Bioactive peptides from whey proteins. Whey Proteins.

[46] Cai C., Zhang Z., Morales M., Wang Y., Khafipour E., Friel J. (2019). Feeding practice influences gut microbiome composition in very low birth weight preterm infants and the association with oxidative stress: A prospective cohort study. Free Radic. Biol. Med..

[47] Cristofalo E.A., Schanler R.J., Blanco C.L., Sullivan S., Trawoeger R., Kiechl-Kohlendorfer U., Dudell G., Rechtman D.J., Lee M.L., Lucas A. (2013). Randomized trial of exclusive human milk versus preterm formula diets in extremely premature infants. J. Pediatr..

